# Comparative effectiveness and safety of herbal medicine therapy for low back pain and radiculopathy caused by lumbar intervertebral disc herniation: a study protocol for a pragmatic randomized controlled trial

**DOI:** 10.3389/fneur.2025.1696098

**Published:** 2025-12-17

**Authors:** Jung-Hyun Kim, Yoon Jae Lee, In-Hyuk Ha, Won-Suk Sung, Eun-Jung Kim, Yeoncheol Park, Yonghyeon Baek, Sang-Soo Nam, Byung-Kwan Seo

**Affiliations:** 1 Department of Acupuncture and Moxibustion Medicine, Kyung Hee University Hospital at Gangdong, Seoul, Republic of Korea; 2 Jaseng Spine and Joint Research Institute, Jaseng Medical Foundation, Seoul, Republic of Korea; 3 Department of Acupuncture & Moxibustion Medicine, Dongguk University Bundang Oriental Hospital, Seongnam-si, Republic of Korea; 4 Department of Acupuncture and Moxibustion Medicine, College of Korean Medicine, Kyung Hee University Hospital at Gangdong, Kyung Hee University, Seoul, Republic of Korea

**Keywords:** lumbar intervertebral disc herniation, herbal medicine, pragmatic randomized controlled trial, traditional Korean medicine, low back pain

## Abstract

**Background:**

Low back pain and radiculopathy due to lumbar intervertebral disc herniation are prevalent conditions, significantly impacting quality of life and imposing substantial socioeconomic burdens globally. In Korea, these conditions are frequently managed within traditional Korean medicine clinics, demonstrating high patient preference for such interventions. However, rigorous clinical evidence on the effectiveness, cost-effectiveness, and safety of individual traditional Korean medicine therapies remains insufficient, particularly for older adult populations where surgical risks are elevated. This highlights a critical need for research into conservative treatment approaches, especially pragmatic randomized controlled trials that reflect real-world clinical practice.

**Methods:**

This pragmatic randomized controlled trial will be a two-arm parallel study designed to evaluate the effectiveness and safety of herbal medicine therapy for low back pain and radiculopathy resulting from lumbar intervertebral disc herniation. Seventy-four eligible adult participants, aged 19 years or older, with magnetic resonance imaging or computed tomography confirmed disc bulging or worse and a Numeric Rating Scale score for radiating pain between three and six, will be randomly assigned to either the herbal medicine strategy treatment group or a usual traditional Korean medicine treatment group that excludes herbal medicine. The primary outcomes will be changes in the Numeric Rating Scale for radiating pain and the Oswestry Disability Index at week seven. Secondary outcomes will include various pain, disability, quality of life, and economic evaluation measures assessed at multiple time points up to 26 weeks. Safety will be continuously monitored through adverse event reporting, laboratory tests, and vital signs. Statistical analysis will primarily use intention-to-treat and per-protocol populations, employing linear mixed models for efficacy endpoints and chi-squared or Fisher’s exact tests for safety data. Non-inferiority testing will be performed if superiority is not established.

**Conclusion:**

This pragmatic randomized controlled trial aims to provide robust clinical evidence on the effectiveness, safety, and cost-effectiveness of herbal medicine therapy for lumbar intervertebral disc herniation, particularly in comparison to other traditional Korean medicine interventions. The findings are expected to contribute significantly to developing evidence-based guidelines and optimizing treatment strategies for this prevalent condition, addressing an urgent healthcare need and improving patient outcomes.

## 1 Background

Low back pain (LBP) and radiculopathy caused by lumbar intervertebral disc herniation (LIDH) represent a significant global health burden that impacts the quality of life and imposes substantial socioeconomic costs (1). In Korea, LIDH is among the most frequently encountered conditions in traditional Korean medicine (TKM) clinics, with high patient preference for TKM interventions (2). Despite its popularity, rigorous clinical evidence on the effectiveness of individual TKM therapies remains insufficient.

LBP and leg pain in middle-aged and older adult populations are frequently associated with LIDH (3). A particular concern arises in older patients, where surgical complications, including those occurring within 3 months post-discharge, are higher in individuals aged ≥65 years compared to younger cohorts (4). Furthermore, studies indicate that among patients aged ≥80 years undergoing lumbar surgery, a notable percentage experience one or more complications or mortality, with a subset experiencing severe complications and a significant 30-day readmission rate (5). These findings underscore the critical need for research on conservative treatment approaches for LIDH, especially in the older adult population.

The escalating socioeconomic burden associated with LIDH necessitates the development of high-quality healthcare services to enhance treatment effectiveness and improve patient satisfaction. This can be achieved by objectively substantiating the efficacy of established TKM therapeutic techniques through clinical trials, thereby identifying optimal treatment strategies tailored to the progression of LIDH (6). However, randomized controlled trials (RCTs) investigating the effectiveness, cost-effectiveness, and safety of individual TKM interventions for LIDH are lacking. Furthermore, standardized clinical pathways for complex TKM treatments of LIDH are yet to be developed and disseminated. This highlights the urgent need for active research and development to address evolving healthcare demands, provide evidence-based care to meet patient needs, and contribute to the advancement of public health.

Treatment strategies for lumbar intervertebral disc herniation (LIDH) fall into conservative and surgical categories. Conservative approaches commonly feature local injections, including facet joint, epidural, and selective nerve root blocks, alongside spinal bracing, bed rest, pharmacotherapy, manipulative therapy, physical rehabilitation, and behavioral interventions. Surgery is typically reserved for severe motor deficits or cauda equina syndrome, with conservative management preferred for the majority of cases (6). TKM offers a range of conservative treatments including acupuncture, Chuna manual therapy, pharmacopuncture, and herbal medicine (7).

Despite numerous studies supporting the efficacy of epidural steroid injections, a considerable number have raised questions about their effectiveness, and a non-negligible incidence of adverse effects has been reported (8–10). The surgical treatment of LIDH, typically involving decompression and fusion, is not without limitations. Reports have suggested that degenerative changes in the intervertebral disc, adjacent segment spondylolisthesis, disc herniation, and spinal stenosis can occur (11). These limitations highlight the need to explore and validate alternative complementary treatment modalities, particularly within TKM, where herbal medicine serves as a foundational modality aimed at addressing internal imbalances through multi-herb formulations, potentially offering distinct advantages in long-term symptom management and holistic recovery compared to procedural interventions like acupuncture, moxibustion, and blood-letting cupping.

This investigation is designed as a pragmatic randomized controlled trial (PRCT) to provide information relevant to decision-making in real-world clinical practice. Unlike explanatory clinical trials, which are typically conducted for drug approval and assess the efficacy of an intervention under ideal conditions, PRCTs evaluate the “overall effectiveness” of a treatment in routine clinical settings (12). It is crucial to understand that PRCTs are not alternatives to traditional randomized clinical trials (RCTs) but rather extensions of explanatory trials (13). Guidelines such as the CONSORT extension checklist and the pragmatic-explanatory continuum indicator summary (PRECIS)-2 are recommended for reporting and evaluating PRCTs (14). A key characteristic of PRCTs is their reflection of actual clinical environments, indicating that treatments and interventions adhere to usual clinical practice, and management methods maintain the same level as real-world care (15).

Numerous clinical studies have adopted this research design, with a notable example being a trial comparing surgical strategies with intensive rehabilitation in patients with chronic LBP. In this study, the PRCT design allowed the use of various surgical methods applicable at each site, determined at the physician’s discretion, without predefining the surgical approach (16). PRCTs are particularly appropriate for evaluating the comparative effectiveness of herbal medicine in treating LIDH, as they enable assessment of interventions under routine clinical conditions and facilitate comparisons where placebo controls are ethically or practically challenging. Despite differences between the experimental arm (herbal medicine, involving daily oral administration) and the control arm (acupuncture, moxibustion, and blood-letting cupping, delivered in periodic sessions), these variations align with standard TKM practice, in which treatment modalities are tailored to individual patient needs and clinical discretion, thereby strengthening the study’s external validity and real-world relevance. To safeguard internal validity and enhance interpretability, the analysis will employ intention-to-treat principles, covariate adjustment for baseline imbalances, sensitivity tests for adherence and frequency variations, and subgroup analyses to explore the influence of modality-specific factors on outcomes, yielding a rigorous and transparent comparison of treatment effects.

## 2 Materials and methods

### 2.1 Study design and objectives

**Figure 1:**
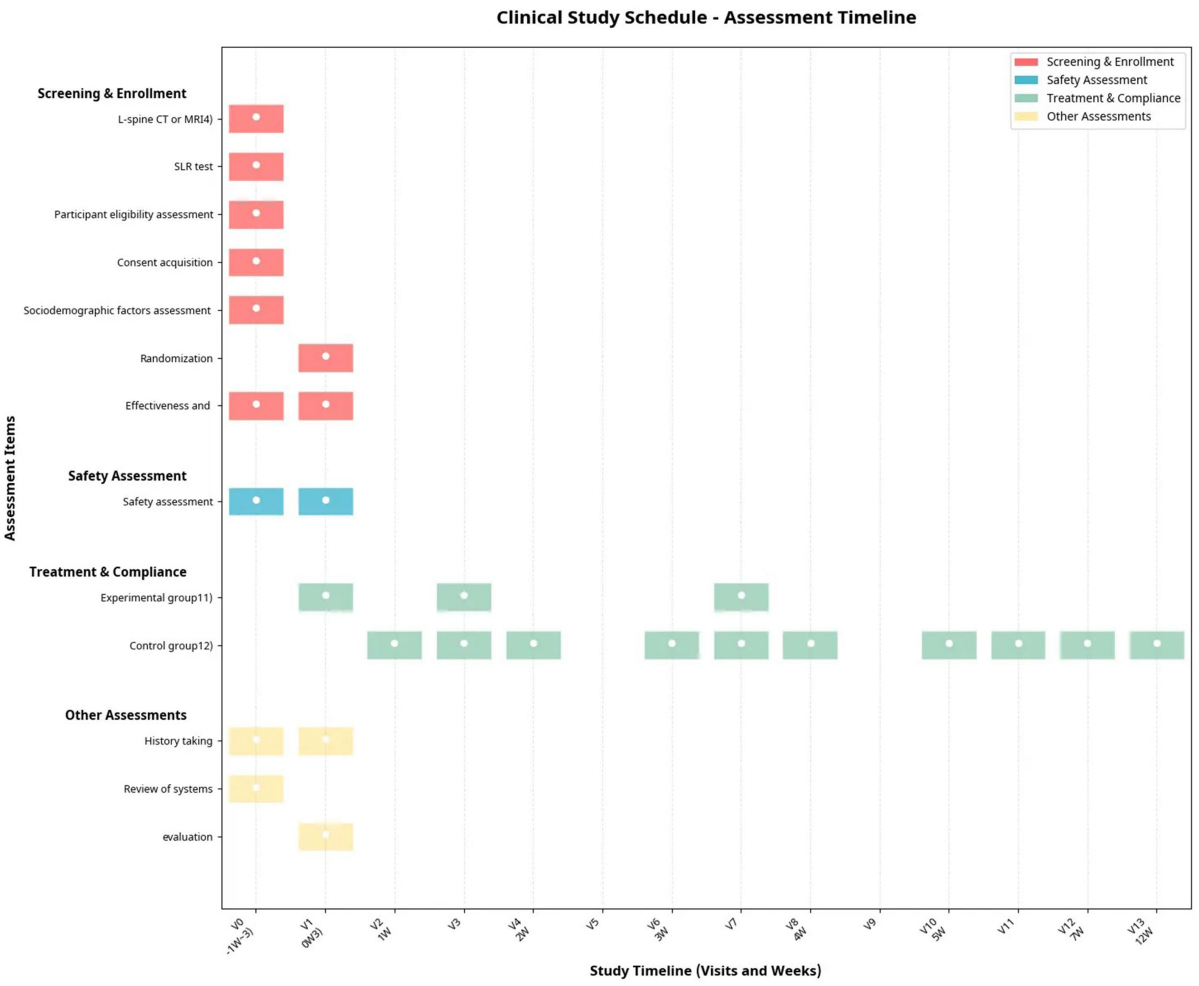
Study flowchart.

This 2-arm parallel pragmatic randomized controlled trial (PRCT) evaluates the effectiveness and safety of herbal medicine versus usual traditional Korean medicine (TKM) interventions, excluding herbal medicine, for low back pain (LBP) and radiculopathy due to lumbar intervertebral disc herniation (LIDH). Eligible patients, meeting predefined inclusion and exclusion criteria, will be randomized to either the herbal medicine or usual TKM group, following a detailed clinical research protocol (see Figure 1).

The primary objective is to compare treatment effectiveness using the Numeric Rating Scale (NRS) for radiating pain and the Oswestry Disability Index (ODI) at week 7 relative to baseline. Secondary objectives include assessing changes in NRS for LBP, 100 mm Pain Visual Analog Scale (VAS), Roland-Morris Disability Questionnaire (RMDQ), EuroQol 5-Dimension 5-Level (EQ-5D-5L), Health Information National Trends Survey (HINT-8), Global Perceived Effect (GPE), and Patient Health Questionnaire-15 (PHQ-15) at weeks 3, 7, 12, 19, and 26, alongside economic evaluations for cost-effectiveness. The Kidney Yin Deficiency Pattern Identification Questionnaire (DSKI) scores will be evaluated at week 7. Treatment expectations, intention to use herbal medicine, and disease-related treatment history will also be assessed. Safety will be monitored through adverse event tracking, clinical laboratory tests, vital signs, body weight, and concomitant medication reviews (see Table 1 for study schedule).

**Table 1:** Study schedule and timelines.

Timelines		Screening	Enrollment allocation	Active treatment post-allocation^2^										Follow-up^1^^,^^2^			
		V0	V1	V2	V3	V4	V5	V6	V7	V8	V9	V10	V11	V12	V13	V14	V15
		−1 W ~ ^3^	0 W^3^	1 W		2 W		3 W		4 W		5 W		7 W	12 W	19 W	26 W
Window period		−14 ~ 0	(baseline)	±4		±4		±4		±4		±4		+14	±7	±7	±7
L-spine CT or MRI^4^		●															
SLR test		●															
Participant eligibility assessment		●															
Consent acquisition		●															
Sociodemographic factors assessment		●															
History taking		●	●														
Review of systems		●												●			
Randomization			●														
Effectiveness and economic evaluation	NRS (lumbago, radiating pain)	●	●					●^5^				●^6^		●	●	●	●
	ODI		●					●^5^				●^6^		●	●	●	●
	100 mm Pain VAS (lumbago, radiating pain)^1^		●											●	●	●	●
	RMDQ		●											●	●	●	●
	EQ-5D-5L		●					●^5^						●	●	●	●
	HINT-8		●					●^5^						●	●	●	●
	GPE							●^5^						●	●	●	●
	PHQ-15		●											●	●	●	●
	Kidney Yin Deficiency Pattern Identification		●											●			
	Economic evaluation^5^		●					●^5^				●^6^		●	●	●	●
	Time costs^5^							●^5^									
	Productivity loss costs^5^		●					●^5^						●	●	●	●
	Treatment expectation scale		●														
	Intention to Choose Herbal Medicine Treatment		●											●			
Safety assessment	Vital signs^1^	●	●					●^5^						●	●	●	●
	Lab tests^7^	●^7^												●^7^			
	Anthropometric measurements	●												●			
	Concomitant medication review^8^	●	●					●				●		●	●	●	●
	Adverse effect assessment^9^			●		●		●		●		●		●	●	●	●
Treatment compliance assessment^10^				▲^11^		▲^11^		▲^11^		▲^11^		▲^11^		▲^11^			
Experimental group^11^			■		■ (10D)			■ (20D)									
Control group^12^				▲	▲	▲	▲	▲	▲	▲	▲	▲	▲				

1 Follow-up visits at weeks 12, 19, and 26 may be conducted via telephone surveys if in-person visits are impractical. In such cases, vital signs and VAS measurements were omitted.

2 Missing follow-up questionnaires (including telephone surveys) due to a participant’s missed visit will not be considered a protocol violation.

3 For the experimental group, weeks −1 and 0 visits were conducted on the same day. For the control group, weeks −1, 0, and 1–1 visits can were conducted on the same day.

4 Lumbar spine CT or MRI examination results, whether performed at the current institution or other hospitals, may be used if previously acquired.

5 At week 3, the assessment of the NRS, VAS, ODI, EQ-5D-5L, HINT-8, GPE, healthcare utilization costs/time survey, time–cost evaluation, productivity loss, and vital signs may have been conducted concurrently with the day 20 visit for the intervention group. For the control group, these assessments will be performed at week 3–1, or week 3–2 if not completed at week 3–1.

6 NRS, ODI assessments, and medical cost surveys at week 5 will be conducted via telephone survey.

7 Participants will undergo blood and urine tests at weeks −1 and 7. They would be required to visit after an 8-h fasting period on the day prior to blood collection for the tests. Retesting for abnormal results may be performed at the discretion of the investigator. However, for week −1 clinical laboratory tests, the results from tests conducted within 14 days of the screening visit can be substituted. The specific test items were as follows: (a) blood test items: WBC, RBC, neutrophils, lymphocytes, monocytes, eosinophils, basophils, MCV, MCH, MCHC, hemoglobin, hematocrit, platelet count, total protein, albumin, ALT, AST, ALP, total bilirubin, gamma GT, uric acid, BUN, creatinine, BUN/creatinine ratio, serum glucose, PT, aPTT, ESR, and CRP. (b) Urine Test Items: specific gravity, pH, protein, glucose, ketones, urobilinogen, bilirubin, blood, nitrite, WBC (leukocytes). For female participants, urine HCG screening was performed at week −1.

8 During the concomitant medication review, both the dosage and frequency of use will be investigated.

9 For the herbal medicine strategy group, adverse events will be confirmed via weekly phone calls starting from week 1 after herbal medicine administration. For TKM treatment group, these assessments were conducted at weeks 1–2, 2–1, 3–1, 4–1, and 5–1. If not conducted during the first visit of the respective week from week 2 to 5, it may be conducted during the second visit of that week.

10 For treatment compliance verification, in the herbal medicine strategy group, the number of days herbal medicine was not taken since receipt was assessed weekly from weeks 1 to 5. At week 7, the total number of prescribed herbal medicine packs and the number of remaining packs were recorded in the CRF. For TKM treatment group, the total number of administered treatments will be recorded in the CRF at week 7.

11 Participants in the herbal medicine strategy group will visit weeks 0, day 10, and day 20 to receive and take a 10-day supply of herbal medicine, totaling 30 days.

12 The frequency of usual TKM treatment will generally adhere to a standard of twice per week but may be adjusted to 1–3 times per week based on the clinician’s judgment regarding the patient’s symptoms, with the total number of sessions not exceeding 10.

### 2.2 Inclusion and exclusion criteria

Participant recruitment for this clinical trial is currently ongoing based on the following pre-specified eligibility criteria. The First Participant Recruitment Date was August 1, 2025. Participating institutions are engaging in competitive recruitment without specific enrollment targets assigned to each site.

#### 2.2.1 Inclusion criteria

Participants will be enrolled if they meet the following criteria:

(1) Male or female adults aged ≥19 years at the time of screening.
(2) Presence of disc bulging or worse in the lumbar spine, confirmed by MRI or CT scans, with clinical correlation to radiating pain symptoms.
(3) Reporting a Numeric Rating Scale (NRS) score of ≥3 and ≤6 for radiating leg pain over the past week, indicating mild to moderate pain suitable for evaluation of traditional alternative therapies as a primary intervention, while not precluding adjunctive conventional treatments where clinically appropriate.
(4) Willingness to participate in the clinical study and provision of written informed consent.

#### 2.2.2 Exclusion criteria

Participants will be excluded if they meet any of the following criteria:

(1) Congenital lumbar spine malformations or lumbar spine surgery within the past 3 months.
(2) Red flag symptoms suggestive of cauda equina syndrome (e.g., bladder/bowel dysfunction, saddle anesthesia).
(3) Lumbar spine co-morbidities, including tumors, fractures, or infections.
(4) Direct lumbar injection therapy (e.g., steroids) within the past week.
(5) Current treatment for psychiatric disorders (e.g., depression, schizophrenia).
(6) Severe medical conditions interfering with treatment or participation (e.g., gastrointestinal, cardiovascular, hypertensive, diabetic, renal, hepatic, or thyroid disorders).
(7) Pregnancy, breastfeeding, or plans to become pregnant.
(8) Contraindications to acupuncture (e.g., bleeding disorders, severe diabetes with infection risk).
(9) Contraindications to herbal medicine, including diseases affecting drug absorption/metabolism, digestive disorders post-surgery, anticoagulant therapy, or severe liver/kidney disease (AST, ALT, *γ*-GTP, or serum creatinine >2x upper normal limit at screening).
(10) Participation in another clinical study within 1 month prior to screening or planned participation during the study or within 6 months post-screening.
(11) Inability to provide written informed consent.

### 2.3 Sample size calculation

The target sample size for this PRCT was rooted on a previous research (17). The required sample size was determined by calculating the minimum number of participants needed to detect a clinically meaningful difference between the intervention and control groups, specifically for the primary outcome measure, using a superiority design. The effect size (Cohen’s d) was estimated at 0.5, representing a moderate difference we aimed to detect. Assuming an alpha level (*α*) of 0.05 (two-sided) and a statistical power (1 − *β*) of 80% (0.80), the required number of participants per group (n) was calculated using the formula for comparing two independent means: *n* = 2 × *δ*2(Zα/2 + Zβ)2 × *σ*2, where Zα/2 and Zβ are the standardized normal deviates corresponding to the significance level and power, σ is the estimated standard deviation, and δ is the minimum clinically important difference. We estimated the standard deviation (σ) to be 10 points and set the minimum detectable difference (δ) to be 5 points on the primary outcome scale. Accounting for an anticipated 15% attrition rate, the total required sample size was adjusted from N_calculated_ to N_final_ to ensure adequate power at the study’s conclusion, resulting in a target enrollment of 74 participants (37 per group). Target sample sizes are listed in Table 2. This trial is powered for a superiority outcome, but a secondary, exploratory non-inferiority (NI) analysis is planned to assess the comparative effectiveness or safety of the intervention if the superiority hypothesis is not met. This approach is clinically relevant for pragmatic trials, evaluating whether the intervention is “not worse” than the control within a predefined NI margin (*Δ*). However, the sample size of 74, calculated for superiority, is likely insufficient for a definitive NI trial, as NI analyses typically require larger samples to ensure the confidence interval of the treatment difference lies within Δ. To address this, the manuscript will clarify that the NI analysis is exploratory and hypothesis-generating, underpowered for this endpoint, and will report the NI margin and two-sided 95% confidence interval for the primary outcome difference.

**Table 2:** Target sample size of the study.

Category	Control group	Intervention group	Total participants
Efficacy evaluable cases	33	33	66
Total sample size (incl. 10% dropout)	37	37	74

### 2.4 Procedures for randomization

An independent statistician will prepare the randomization list in SAS version 9.1.3 (SAS Institute Inc., Cary, NC, United States), using a fixed seed to ensure reproducibility. At visit 1, eligible participants—those satisfying the inclusion and exclusion criteria—will be assigned to either the herbal medicine or standard traditional Korean medicine (TKM) group through block randomization with a 1:1 ratio. Each participant will receive a site-specific enrollment number and a randomization code delivered via sealed envelopes handled by an external party. During screening, investigators will secure informed consent, document eligibility, and issue consecutive screening codes (e.g., S1-01). Once eligibility and consent are verified, participants will be enrolled and given a unique allocation code (e.g., R1-01) linked to the randomization schedule.

This open-label study maintains assessor blinding: outcome evaluators (research nurses or residents), who neither provide treatment nor know group assignments, will perform assessments in isolated rooms. Items at risk of unblinding—such as credibility/expectancy scales, cost records, medication logs, and adverse event reports—are exempt from this blinding.

### 2.5 Interventions

This pragmatic randomized controlled trial will assign participants to either the herbal medicine or TKM treatment group. The specific methods for both herbal medicine and typical TKM interventions will not be predefined; instead, they will be determined by the clinician’s judgment based on the patient’s individual condition and progress, with all administered interventions meticulously recorded in case report forms (CRFs).

#### 2.5.1 Herbal medicine

This strategy focused on evaluating the effectiveness of herbal medicines. The type of herbal formula and its administration will be selected from those commonly prescribed for lumbar intervertebral disc herniation in acupuncture and moxibustion textbooks of TKM (18). Dosage instructions will be provided by the clinician and details such as formula type, administration frequency, and pack volume will be recorded. Herbal medicines are prepared at the institution’s dispensary for patient pickup or delivery. Compliance will be monitored weekly, and at week 7, the total number of prescribed and remaining packages will be counted. Participants in this group will take herbal medicines orally for 30 days, with clinic visits every 10 days for follow-ups and new prescriptions, totaling three 10-day prescriptions.

#### 2.5.2 Usual traditional Korean medicine treatments

This strategy employs a combination of traditional Korean medicine (TKM) therapies for lumbar intervertebral disc herniation, selected by a Korean medical doctor based on patient symptoms and progress. Modalities include acupuncture (e.g., BL23, BL25, GV3, GV4, BL32, BL40, BL60, GB30, GB34, SP6 for 15 min), electroacupuncture (BL23, BL25 for 15 min at 3 Hz), transcutaneous infrared irradiation, bloodletting cupping (L1-L5 for 5 min), moxibustion (43 ± 1 °C on two acupoints for 15 min), and physical therapy (ICT or TENS for 10–15 min). Treatments occur twice weekly for 5 weeks, adjustable to 1–3 times per week based on patient needs, with a maximum of 10 sessions. All treatment details are recorded.

#### 2.5.3 Concomitant therapy

To ensure that the study reflects real-world clinical practice, there will be no restrictions on concomitant therapies for LBP or radiculopathy symptoms during the study period. All concurrent treatments were fully documented for accurate evaluation.

### 2.6 Outcome measures

This study used a comprehensive set of outcome measures to evaluate the effectiveness and safety of the interventions. All patient-reported outcomes were assessed at specific visits throughout the study.

(1) NRS scores for pain intensity (low back and radicular pain).

At weeks −1 (baseline), 0, 3, 5, 7, 12, 19, and 26, participants will assess their low back pain and radiating leg pain over the past week using an 11-point Numeric Rating Scale (NRS), ranging from 0 (no pain) to 10 (worst pain imaginable), selecting the number that best reflects their current discomfort level.

(2) Oswestry disability index.

At weeks 0, 3, 5, 7, 12, 19, and 26 visits, participants will complete the ODI (19) questionnaire by providing answers to each question.

(3) VAS score for pain intensity (low back and radicular pain)

At weeks 0, 7, 12, 19, and 26, participants will use a 100 mm Pain Visual Analog Scale (VAS) (20) to quantify their LBP experienced over the past week. Participants will indicate their current pain level by marking a point on a 100 mm Visual Analog Scale (VAS), ranging from “no pain” to “worst pain imaginable”.

(4) Roland-Morris disability questionnaire (RMDQ)

At weeks 0, 7, 12, 19, and 26, the participants will complete the Roland-Morris Disability Questionnaire (RMDQ) (21) by answering each question.

(5) EuroQol-5 dimension-5 level (EQ-5D-5L)

At Weeks 0, 3, 7, 12, 19, and 26, the participants will complete the EQ-5D-5L (22) questionnaire by responding to each question.

(6) Health-related quality of life instrument with eight items (HINT-8)

At weeks 0, 3, 7, 12, 19, and 26, participants will complete the HINT-8 questionnaire (23). Participants will assess their health status over the past week by selecting one of four response levels for each question, ranging from 1 (e.g., “not at all” for climbing stairs or “not at all depressed” for depression) to 4 (e.g., “unable to climb” or “always depressed”).

(7) Global perceived effect (GPE)

At weeks 3, 7, 12, 19, and 26, participants completed the Global Perceived Effect (GPE) questionnaire (24) by providing answers to each question.

(8) Patient health questionnaire-15 (PHQ-15)

At weeks 0, 7, 12, 19, and 26, participants will complete the Korean version of the Patient Health Questionnaire-15 (PHQ-15). This tool, a 15-item condensed version (25) developed by Kroenke et al. from Spitzer et al.’s PHQ (26), assesses general health status and subjective somatic symptoms.

(9) Kidney Yin deficiency pattern identification questionnaire score (DSKI)

At weeks 0 and 7, participants will complete the Kidney Yin Deficiency Pattern Identification Questionnaire (DSKI) (27) to assess the Kidney Yin Deficiency Pattern, a diagnostic construct in Korean traditional medicine characterized by symptoms such as lower back pain, fatigue, night sweats, dry mouth, and insomnia, reflecting an imbalance in yin energy essential for bodily nourishment and cooling. The DSKI quantifies these symptoms through a standardized set of questions, with scores indicating the severity of the deficiency pattern. This measure is relevant to the study as it evaluates the impact of traditional alternative therapies on a key diagnostic pattern associated with lumbar disc-related radiating pain.

(10) Intention to choose herbal medicine.

At weeks 0 and 7, participants were asked about their perceived need for herbal medicine treatment in their current condition.

(11) Treatment expectation scale.

At the initial visit, participants will rate their expectations of symptom relief from herbal medicine and acupuncture treatments on a 9-point Likert scale (1 = not at all, 5 = somewhat, 9 = very much).

### 2.7 Statistical analysis

Data will be analyzed using intention-to-treat (ITT) and per-protocol (PP) sets. The ITT population includes all randomized participants receiving at least one dose of herbal medicine or one traditional Korean medicine (TKM) session. The PP population comprises ITT participants with ≥70% herbal medicine compliance or ≥7 TKM sessions. Efficacy outcomes will be assessed in both ITT and PP populations, with ITT as the primary analysis and PP as supportive. Demographic and safety evaluations will use the ITT population, with safety analyses based on raw data without imputation. Statistical analyses will be conducted using SAS version 9.4 (SAS Institute, Cary, NC, United States) with a two-sided significance level of 0.05 and 95% confidence intervals.

#### 2.7.1 Analysis of sociodemographic data and treatment expectations

Sociodemographic characteristics and treatment expectations will be assessed, with data stratified by age (younger adults: <65 years; older adults: ≥65 years) to account for potential variations in acceptance and efficacy of herbal medicine across age groups. Continuous variables will be reported as mean ± standard deviation or median (interquartile range) and compared using Student’s t-test or nonparametric tests for non-normal data, with age-stratified subgroup analyses. Categorical variables will be presented as frequencies (%) and compared using chi-squared or Fisher’s exact test, incorporating age stratification to enhance generalizability.

#### 2.7.2 Analysis of efficacy endpoints

1) Primary efficacy endpoint

Changes in Numeric Rating Scale (NRS) for radiating pain and Oswestry Disability Index (ODI) from baseline to week 7 will be analyzed using a Linear Mixed Model, with group as a fixed factor and relevant baseline covariates. Missing data will be handled via Mixed Model for Repeated Measures (MMRM), with sensitivity analyses using ANCOVA on datasets imputed by multiple imputations (MI) and Last Observation Carried Forward (LOCF).

2) Secondary efficacy endpoints

Changes in NRS for low back pain, 100 mm Pain Visual Analog Scale (VAS), Roland-Morris Disability Questionnaire (RMDQ), EuroQol 5-Dimension 5-Level (EQ-5D-5L), Health Information National Trends Survey (HINT-8), Global Perceived Effect (GPE), Patient Health Questionnaire-15 (PHQ-15), economic measures, and Kidney Yin Deficiency Pattern Identification Questionnaire (DSKI) scores will be analyzed using a Linear Mixed Model with baseline covariates. Missing data will be managed via MMRM, with ANCOVA sensitivity analyses using MI and LOCF. Non-inferiority testing, with margins of −1.75 (NRS) and −10 (ODI), will be conducted if superiority is not achieved, considering herbal medicine non-inferior if the 95% confidence interval lower bound does not exceed these margins.

#### 2.7.3 Analysis of safety endpoints

Adverse events (AEs), including those leading to withdrawal and serious AEs, will be summarized by treatment group and compared using chi-squared or Fisher’s exact test. Incidence rates of all AEs and herbal medicine-specific AEs will be reported.

#### 2.7.4 Cost-effectiveness analysis

Economic evaluation will adopt a societal perspective, using a micro-costing approach to compare intervention and control groups over the follow-up period. The Incremental Cost-Effectiveness Ratio (ICER) will be calculated as the cost difference (ΔC) divided by the Quality-Adjusted Life Years (ΔE) difference from EQ-5D-5L scores. Costs include direct medical (e.g., procedures, hospitalization), direct non-medical (e.g., transportation), and indirect costs (e.g., productivity loss). Non-parametric bootstrapping (1,000 replications) will estimate ICER confidence intervals, presented on a Cost-Effectiveness Plane, with a Cost-Effectiveness Acceptability Curve illustrating cost-effectiveness probabilities across willingness-to-pay thresholds.

#### 2.7.5 Missing value management

Missing data for primary and secondary outcomes will be managed using LOCF imputation, selected for its conservative approach in estimating treatment effects post-dropout, assuming stable participant conditions after the last observation, ensuring robust and interpretable efficacy results.

### 2.8 Dropout and withdrawal

Participants may withdraw voluntarily at any point or be removed by the investigator, with the main reason recorded. Those exiting before treatment begins count as screening failures and, barring medical need, need not complete discontinuation evaluations. Withdrawals due to adverse events (AEs) will receive suitable care, and monitoring will persist for intervention-related AEs until resolved or stabilized. Exclusion criteria include: (1) participant or legal representative requesting withdrawal or revoking consent; (2) newly identified conditions impacting study results; (3) major breaches of inclusion/exclusion rules; (4) confirmed pregnancy; (5) a serious AE necessitating withdrawal or making involvement impractical; (6) barriers to conducting medical or traditional Korean medicine (TKM) procedures for lumbar disc herniation; (7) investigator judgment that ongoing participation is unsuitable; or (8) herbal medicine adherence below 70% across 5 weeks or fewer than six usual care sessions attended. Follow-up assessments may proceed with consent after treatment ends.

### 2.9 Safety

The investigator will evaluate and document all adverse events (AEs) and serious adverse events (SAEs) during the study, adhering to WHO intensity classification guidelines, with non-covered AEs graded as: grade 1 (mild, transient, no treatment or activity limitation); grade 2 (moderate, mild to moderate activity limitation, may require treatment and recovery); or grade 3 (severe, marked limitation, requiring medical intervention or hospitalization). AEs and SAEs will be recorded in the Case Report Form (CRF) using medical terminology or detailed symptoms/signs, with pre-existing conditions noted in screening history and pre-treatment AEs documented separately. Emergency responses include oral NSAIDs for sudden pain exacerbation and immediate cessation, first aid, and emergency referral for acupuncture-related AEs (e.g., pneumothorax, bleeding, fainting). SAEs, except minor gastrointestinal discomfort (e.g., heartburn from herbal medicine), will be reported to the principal investigator or coordinator per regulations within 30 days post-trial. The CRF AE log will capture symptoms, onset, intensity, course, actions, outcome, and causality, with ongoing AEs reviewed, resolved AEs updated, escalating AEs logged anew, and treatment adjustments documented.

### 2.10 Data management

Quality control (QC) and data management protocols will safeguard the accuracy, integrity, and confidentiality of trial data. Secure, password-protected electronic Case Report Forms (eCRFs) within a validated electronic data capture (EDC) system—equipped with range checks and logical constraints—will facilitate data collection while reducing entry errors. All research staff will receive standardized training in study protocols, data acquisition, and QC procedures. Source data verification (SDV) will encompass 100% of primary endpoint data and 10% of secondary endpoint data to confirm alignment with original records. Data will reside on a secure, firewalled server featuring nightly automated backups, with access restricted to authorized individuals through a rigorous control matrix that permits read-only privileges for analysts and full privileges solely for the principal investigator and data manager.

## 3 Discussion

LIDH presents a complex clinical challenge, driving significant patient preference for diverse treatment modalities, including TKM (28). Despite this established patient inclination, robust clinical evidence, particularly from PRCTs, remains crucial for objectively validating the effectiveness and safety of individual TKM interventions for LIDH (29). This study outlines a PRCT specifically designed to assess the comparative benefits of incorporating herbal medicines into a comprehensive TKM treatment strategy for LIDH-related LBP and radiculopathy.

The selection of a PRCT design is a deliberate choice that acknowledges the inherent complexities and individualized nature of TKM. Unlike explanatory trials that prioritize internal validity under tightly controlled conditions, PRCTs aim to maximize external validity by reflecting real-world clinical practice (30). This design enabled assessment of the interventions’ overall effectiveness—a critical metric in TKM, where regimens adapt dynamically to individual symptoms and progress. By contrasting a herbal medicine-inclusive group with a standard TKM cohort (herbal medicine excluded), the study precisely measures herbal contributions within real-world TKM practice. Clinician discretion in modality selection, guided by expertise, enhances the results’ generalizability and relevance to routine care.

This trial addresses a notable gap in the existing literature by directly comparing herbal medicine strategies with other established TKM interventions. While previous studies have explored various TKM modalities for LBP, including acupuncture (31) and pharmacopuncture (32), and some PRCTs have contrasted TKM with conventional medicine, comprehensive PRCT focusing on the strategic role of herbal medicine in LIDH are scarce. A comprehensive suite of patient-reported outcomes—encompassing pain intensity, functional disability, quality of life, and global perceived effects—coupled with rigorous safety monitoring and cost-effectiveness assessments, generated robust evidence. These data inform clinical decisions, refine patient management, and may underpin evidence-based guidelines for integrated care of lumbar intervertebral disc herniation (LIDH). The predefined statistical plan, which handled missing data and pragmatic design features, bolstered the credibility and clarity of results, advancing insight into herbal medicine’s contribution to LIDH treatment.

Nevertheless, limitations persist. Enrolling patients with disc pathology of “bulging or worse” captured real-world TKM presentations of neurocompressive radiculopathy and axial pain yet compromised cohort uniformity, potentially attenuating effect-size precision and complicating interpretation across severity strata. Subgroup analyses stratified by radiological grade will temper this concern and strengthen applicability to routine practice. The open-label format, inevitable in pragmatic research, invited performance and detection biases from treatment awareness; furthermore, the modest sample (*n* = 74), powered for superiority, constrained non-inferiority exploration. Blinded outcome assessment, intention-to-treat, and per-protocol analyses mitigated these risks, though larger trials incorporating placebo arms remain warranted. Finally, absence of fixed treatment algorithms fostered clinical variability, risking dilution of intervention-specific signals and internal validity. Subsequent investigations should enforce protocol standardization, mandate granular documentation of therapeutic rationales, and deploy techniques such as propensity score matching to sharpen causal inference and broaden generalizability.

## 4 Ethics approval and consent to participate

This study adheres to ethical guidelines to ensure voluntary participation and protect vulnerable populations, defined as those susceptible to undue influence (e.g., medical/pharmacy students, healthcare workers, pharmaceutical employees, military personnel) or with limited autonomy (e.g., individuals with incurable diseases, institutionalized, unemployed, impoverished, ethnic minorities, homeless, refugees, minors, or unable to provide informed consent). Special protections are afforded to these groups, and participation by clinical research institution employees is confirmed as voluntary through handwritten statements. Given the prevalence of lumbar intervertebral disc herniation in older adults (≥70 years), the exclusion criterion of inability to provide written informed consent is strictly enforced. Participants unable to read consent materials will have them read by an impartial third-party witness, and investigators will explain the consent form clearly and slowly to ensure informed, voluntary participation.

The study protocol received ethical approval from multiple Institutional Review Boards (IRBs) at Kyung Hee University Hospital in Gangdong (IRB No. KHNMCOH 2024–05-001, 25 September 2024), Dongguk University Bundang Oriental Medicine Hospital (IRB No. 2024–006, 4 November 2024), and Jaseng Hospital of Korean Medicine (IRB No. JASENG202410005-HE001, 20 November 2024), and Bucheon Jaseng Hospital of Korean Medicine (IRB No. JASENG202410004001-HE001, 20 November 2024). This protocol is officially registered with the Clinical Research Information Service (CRIS) of the Korea Disease Control and Prevention Agency (KDCA), National Institute of Health, under registration number KCT0010035 (Registration date: 25 September 2024).
